# Qualitative and Quantitative Analyses of the Echolocation Strategies of Bats on the Basis of Mathematical Modelling and Laboratory Experiments

**DOI:** 10.1371/journal.pone.0068635

**Published:** 2013-07-05

**Authors:** Ikkyu Aihara, Emyo Fujioka, Shizuko Hiryu

**Affiliations:** 1 Brain Science Institute, RIKEN, Wako, Saitama, Japan; 2 Faculty of Life and Medical Sciences, Doshisha University, Kyotanabe, Kyoto, Japan; National Research & Technology Council, Argentina

## Abstract

Prey pursuit by an echolocating bat was studied theoretically and experimentally. First, a mathematical model was proposed to describe the flight dynamics of a bat and a single prey. In this model, the flight angle of the bat was affected by 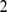 angles related to the flight path of the single moving prey, that is, the angle from the bat to the prey and the flight angle of the prey. Numerical simulation showed that the success rate of prey capture was high, when the bat mainly used the angle to the prey to minimize the distance to the prey, and also used the flight angle of the prey to minimize the difference in flight directions of itself and the prey. Second, parameters in the model were estimated according to experimental data obtained from video recordings taken while a Japanese horseshoe bat (*Rhinolphus derrumequinum nippon*) pursued a moving moth (*Goniocraspidum pryeri*) in a flight chamber. One of the estimated parameter values, which represents the ratio in the use of the 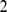 angles, was consistent with the optimal value of the numerical simulation. This agreement between the numerical simulation and parameter estimation suggests that a bat chooses an effective flight path for successful prey capture by using the 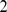 angles. Finally, the mathematical model was extended to include a bat and 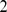 prey. Parameter estimation of the extended model based on laboratory experiments revealed the existence of bat’s dynamical attention towards 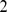 prey, that is, simultaneous pursuit of 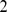 prey and selective pursuit of respective prey. Thus, our mathematical model contributes not only to quantitative analysis of effective foraging, but also to qualitative evaluation of a bat’s dynamical flight strategy during multiple prey pursuit.

## Introduction

Animals have various sensory systems to localize targets such as prey and conspecifics. In general, sensory systems, i.e., visual, olfactory, and auditory organs, *passively* detect information originating from such targets. For example, zebra finches have visual organs that allow them to detect ultraviolet wavelengths [1]; male silkmoths sense olfactory information that is unique to the sex pheromones of conspecific females [2], [3]; barn owls precisely estimate time differences in the arrival of sounds generated by prey during darkness [4]; and male concave-eared torrent frogs detect ultrasound to acoustically interact with conspecific males [5]. Moreover, animals pursue moving targets with high accuracy, using such unique sensory systems. For instance, male houseflies use visual information on conspecifics to chase other males [6], while dragonflies steer to minimize the movement of the image of prey on their retina, and directly fly towards the point of interception [7], [8].

Only a few species of animals, e.g., whales and bats, capture prey by *active sensing*, namely, by emitting ultrasound pulses as sensing signals and detecting the echoes reflected from the prey [9]–[11]. The echolocating behavior of bats has been studied on the basis of field research and laboratory experiments. Field research has shown that bats exhibit high performance during prey pursuit in natural habitats [12]–[14]; the bats dynamically change not only the acoustical characteristics of the ultrasound pulses, but also their flight paths to approach prey. Laboratory experiments have demonstrated further details of active sensing by bats, e.g., patterns of gaze angles under controlled flight tasks [15]–[18]. These behavioral experiments suggest that the bats exhibit a unique flight strategy during prey pursuit. To theoretically evaluate the efficiency of the flight strategy during prey pursuit, mathematical modelling as dynamical systems can be helpful. A number of theoretical studies has used dynamical models to evaluate the behavior of moving animals, such as bird flocks and fish schools [19], [20]. However, the echolocating behavior of bats has not been sufficiently investigated using dynamical models.

In the present study, we performed numerical simulations to theoretically calculate the success rate of prey capture by an echolocating bat. We then estimated the parameters of the mathematical model, based on experiments using Japanese horseshoe bats (*Rhinolphus derrumequinum nippon*) and moths (*Goniocraspidum pryeri*) in a flight chamber.

## Methods

### Mathematical Modelling of Prey Pursuit by an Echolocating Bat

**Figure 1:**
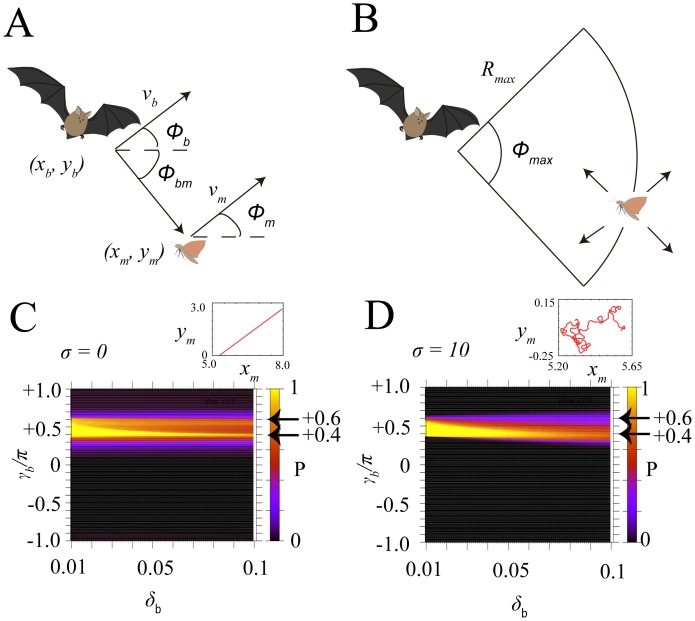
A mathematical model of a bat pursuing a single prey. (A) Definitions of the variables and parameters used in the model. (B) Echolocation range used in the numerical simulation. 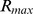 and 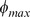 represent the maximum distance and angle at which a bat can detect echoes. (C) The success rate of prey capture, 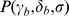, numerically calculated in 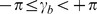, 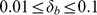 and 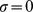, and an example of a prey path at 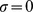 (inset). (D) The success rate of prey capture, 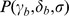, in 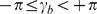, 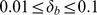 and 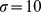, and an example of a prey path at 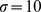 (inset). 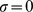 corresponds to the linear motion of prey, and 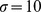 corresponds to the random motion of the prey. 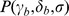 has a high value almost everywhere within 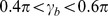. In addition, 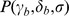 has a higher value, i.e., almost 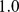, around 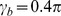. These numerical simulations were performed under the assumption of 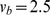 m/s, 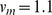 m/s, 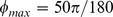 rad, 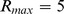 m, and 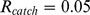 m, which were estimated from experimental data.

Experimental studies in a flight chamber previously revealed that the positions of bats and moths changed much greater in the horizontal plane of the chamber than those did in the vertical plane [18], [21]; changes in the horizontal plane exceeded 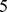 m, whereas those in the vertical plane were less than 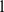 m. Therefore, in the present study, we focus on changes in the positions of a bat and a single prey in the horizontal plane, and model their flight dynamics as follows:
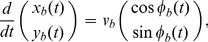
(1)

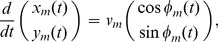
(2)where (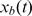, 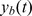) and (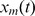, 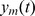) represent the positions of the bat and prey in the horizontal plane, respectively; 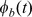 and 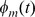 are the flight angles of the bat and prey; and the parameters 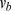 and 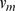 are the flight velocities of the bat and prey. For simplicity, the bat and prey are assumed to fly with constant velocities 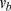 and 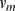. In addition, 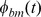 is defined as the angle from the bat to the prey, as shown in Figure 1A.

An echolocating bat can detect differences in the time and sound pressure level of arrivals of echoes reflected from moving prey, by using its 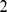 ears. Whereas these differences include information about the angle from the bat to the prey, the time difference between pulse emission and echo arrival includes information about the distance from the bat to the prey. Therefore, the bat can localize the prey by a single pulse in theory. In addition, the bat successively emits ultrasound pulses, allowing it to roughly estimate the flight angle of the prey. Hence, it is assumed that the bat can use 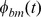 to minimize the distance from itself to the prey, and also 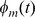 to minimize the difference in flight directions of itself and the prey. Then, the prey is considered to fly with linear or random motion, to numerically simulate its various flight paths. Consequently, the dynamics of 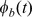 and 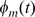 are modeled as follows:
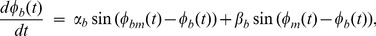
(3)

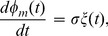
(4)where



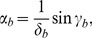
(5)

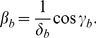
(6)


The parameters 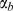 and 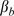 describe how rapidly the bat changes 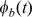, depending on the angular differences of 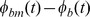 and 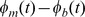, respectively. 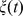 represents white noise, satisfying 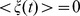 and 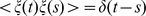 at time 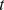 and 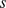. The parameter 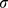 describes the intensity of the noise. As shown in Figure 1C and D, 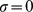 corresponds to the linear motion of the prey, and 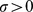 corresponds to the random motion of the prey. Equations 5 and 6 define the relationships of 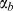 and 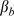 with 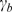 and 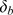; 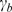 varies from 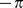 to 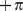 and gives the ratio of 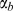 and 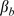, while 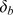 is a positive weighting factor common to 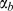 and 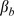. These parameter values, i.e., 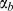, 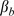, 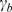 and 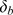, can be determined by fitting the model to experimental data on flight paths of a bat and a prey.

Let us consider 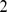 simple cases for 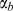 and 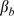, to explain why a sinusoidal function is used in Equation 3. The first case (Case 1) is (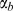, 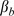) 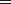 (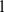, 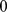), and the second case (Case 2) is (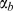, 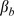) 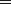 (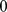, 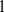). In Case 1, the second term on the right side of Equation 3 is equal to 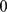, and does not affect the dynamics of 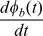. Then, the sign of 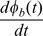 depends on 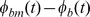: namely, 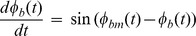 is negative (or positive), when 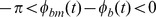 (or 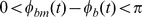). This means that, when the prey is located to the right (or left) of the bat’s flight direction, the bat changes 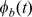 clockwise (or counter-clockwise) and approaches the prey. In Case 2, the first term on the right side of Equation 3 is equal to 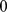. Then, the sign of 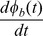 depends on 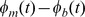: namely, 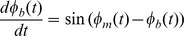 is negative (or positive), when 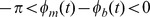 (or 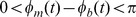). This means that, when the flight direction of the prey is to the right (or left) of the bat’s flight direction, the bat changes 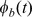 clockwise (or counter-clockwise) and flies in the same direction as the prey. These properties of a sinusoidal function are useful for modeling our assumption that an echolocating bat uses 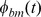 and also 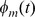, to determine its own flight angle of 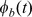, during prey pursuit.

## Results

### Numerical Simulation of the Success Rate of Prey Capture

The success rate of prey capture by a bat is calculated as follows:

- **Initial Conditions.** The parameters 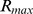 and 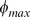 represent the maximum distance and angle for the bat to detect echoes from the prey (Figure 1B). A bat is located at the origin in the 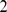-dimensional space, and then starts to fly towards the right, i.e., 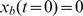, 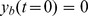, and 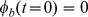. A single prey is located on an edge of bat’s echolocation range, i.e., 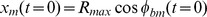, 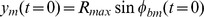 with 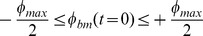, and starts to fly towards a random direction of 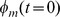 between 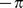 and 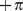.
- **Conditions of Prey Capture.** The parameter 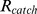 describes the distance within which the bat can capture the prey. If the prey moving with linear (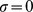) or random (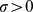) motion escapes from the echolocation range constrained by 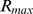 and 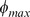, the case is considered a fail. If the prey remains within the echolocation range and is approached by the bat within 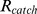, the case is considered a success.

The parameters 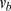, 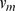, and 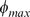 were estimated by using experimental data previously obtained from video and sound recordings in a flight chamber [18], [21]. In the experiments, a moth (*Goniocraspidum pryeri*) was tethered to the ceiling of the chamber (length 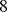 m; width 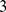 m; height 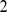 m) by using a piece of string; a bat (*Rhinolphus derrumequinum nippon*) then approached the fluttering moth [18], [21]. The flights of the bat and moth were recorded by 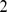 high-speed cameras (MotionPro X3, Integrated Design Tools, Inc., Florida, USA) capturing 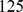 frames per second, as well as a 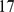
ch horizontal microphone array system with a sampling frequency of 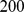 kHz. Based on the video recordings obtained from 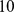 sessions of successful prey capture by 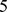 bats, the flight paths of the bats and moths were reconstructed in the 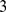-dimensional space of the chamber. The average flight velocities of the bats and moths in the horizontal plane of the chamber were 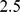 m/s and 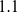 m/s, and therefore 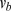 and 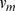 in Equations 1 and 2 were assumed to be 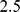 m/s and 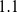 m/s, respectively. In addition, the horizontal angle at which the maximum sound pressure level of the pulses decayed by 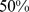 was 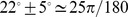 rad from the pulse direction of the bats [18], so that 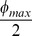 was assumed to be 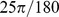 rad. Regarding 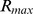, experiments using several species of prey such as midges and caddisflies revealed the maximum echolocation distance by 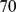
kHz ultrasound as about 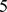
m [22]. In our experiments, the dominant frequency emitted by the bats was 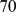 kHz [18], [21], and the wing span of the moths (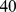
–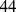
mm) was longer than that of the midges and caddisflies used in [22]. Consequently, the sound pressure level of the echoes reflected from the prey in our experiments were likely larger than those reflected from the midges and caddisflies. Therefore, the bats (*Rhinolphus derrumequinum nippon*) could locate the moths (*Goniocraspidum pryeri*) far from 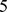 m in our experiments, and then 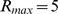 was assumed as the shortest echolocation range in the numerical simulation. Furthermore, the mean body length of the bats was 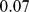 m [18], so that each bat could capture a moth within 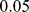 m of itself, i.e., 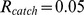 in the numerical simulation.

Under the assumption of 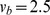 m/s, 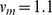 m/s, 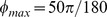 rad, 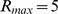 m, and 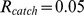 m, the success rate of prey capture was numerically calculated as 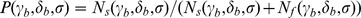, where 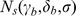 and 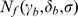 represent the numbers of successful and failed prey capture for each parameter set of 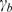, 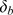 and 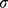. The initial conditions of 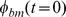 and 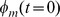 were varied in 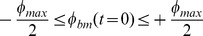 and 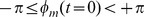 at the interval of 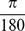
rad.

Figure 1C and D shows the results of the numerical simulation: (C) 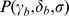 in 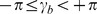, 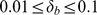 and 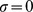 (corresponding to the linear motion of the prey); and (D) 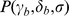 in 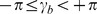, 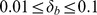 and 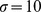 (corresponding to the random motion of the prey). The numerical simulation for 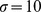 was performed using the Euler-Maruyama method [23] with a time step of 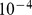. It is shown that 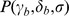 takes a high value almost everywhere within 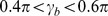. Equations 5 and 6 with 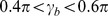 give a positive 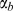, satisfying 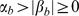 (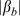 is equal to 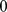 only in the case of 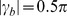), where 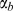 represents the effect of 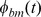 on 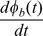 as shown in Equation 3. Hence, high 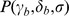 within 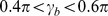 means that, if the bat uses mainly 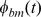 but also 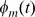 to determine 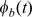, the bat can successfully capture its prey.

Furthermore, 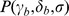 takes a higher value, i.e., almost 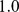, around 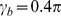 (Figure 1C and D). Equations 5 and 6 with 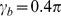 give 
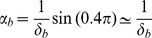
 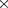 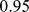 and 
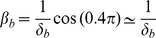
 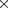 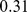, where 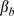 represents the effect of 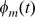 on 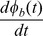 as shown in Equation 3. Therefore, the higher value of 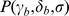 around 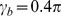 means that, if a bat uses 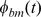 and 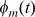 approximately in the ratio of 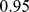 to 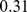, the bat can capture its prey more successfully. Thus, the flight angle of the prey, 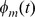, is also important for the more successful capture of a single prey by a bat.

In summary, by performing the numerical simulation with the present mathematical model, we have demonstrated that 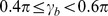 is a suitable parameter value for an echolocating bat to capture a single prey in a flight chamber.

### Parameter Estimation: The Pursuit of a Single Moth by a Bat

Based on the flight paths of the bats (*Rhinolphus derrumequinum nippon*) and the moths (*Goniocraspidum pryeri*) in the 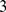-dimensional space of the flight chamber previously examined by laboratory experiments [18], [21], the values of 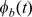, 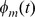, and 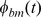 in Equation 3 were estimated as follows:
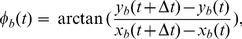
(7)

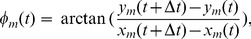
(8)

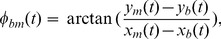
(9)where (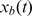, 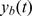) and (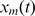, 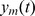) represent the flight paths of a bat and a moth in the horizontal plane of the chamber. The parameter 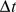 represents a time step of the video recordings at 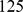 frames per second [18], [21], i.e., 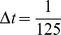
s. The value of 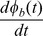 in Equation 3 was estimated as follows:



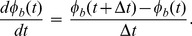
(10)Using the time series data for 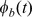, 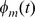, 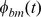, and 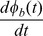 obtained from the laboratory experiments, the parameters 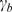 and 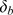 in Equations 5 and 6 were calculated. First, Equation 3 was transformed to 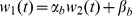 with 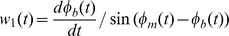 and 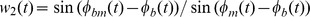. Second, the parameters 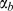 and 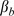 in Equation 3 were estimated at each time 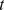, by applying the least-squares method to the neighboring 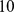 sets of 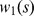 and 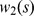 with 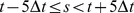. Finally, the parameters 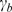 and 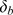 were then calculated using Equations 5 and 6.

**Figure 2:**
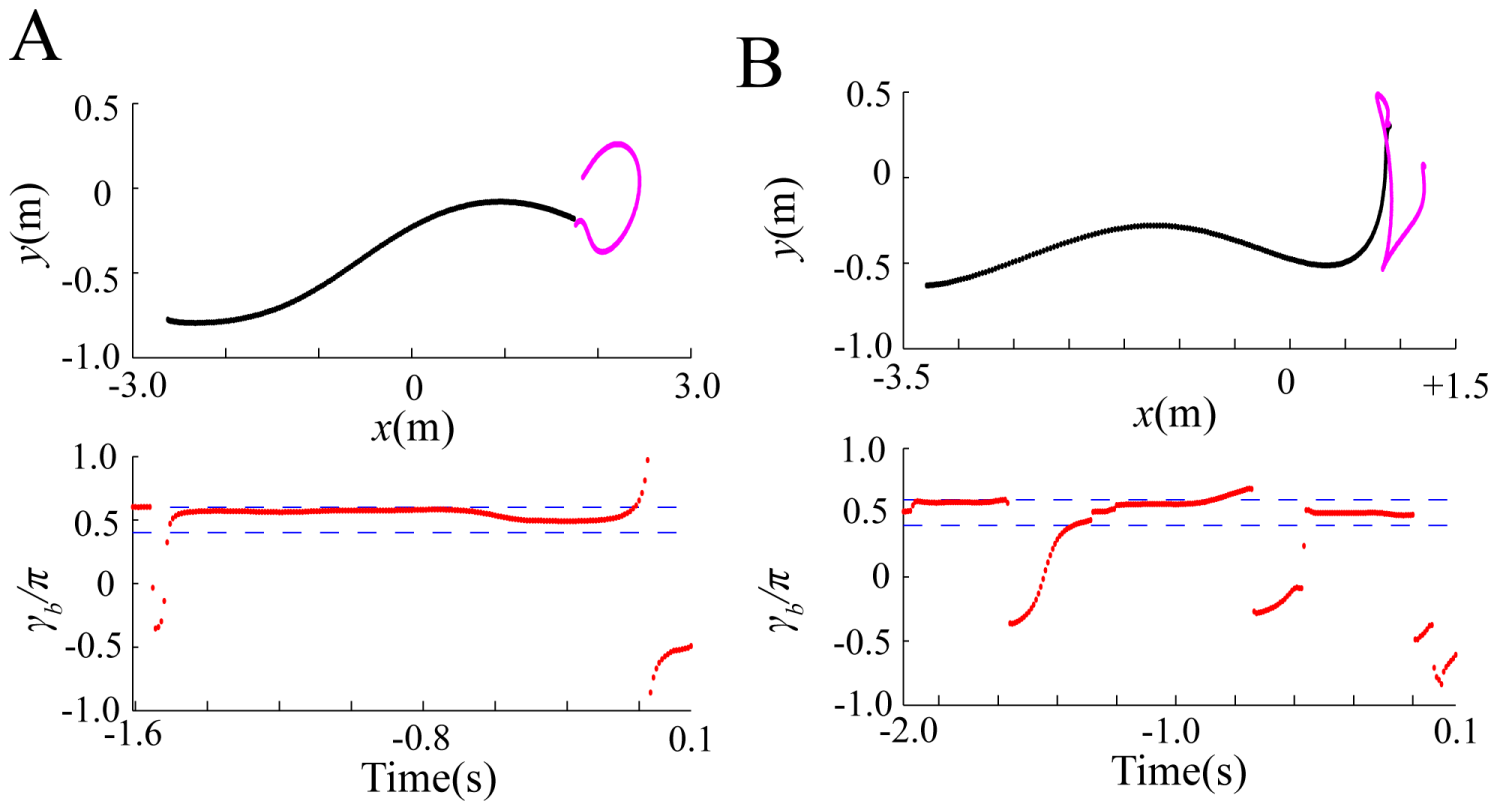
Parameter estimation of 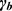 in Equations 5 and 6 from 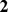 different flight paths (A and B) of a bat and a moth. These 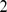 paths were experimentally obtained from video recordings in a flight chamber, using different bat and moth individuals. The top panels represent the flight paths of a bat and a moth in the horizontal plane of the chamber, where the paths of the bat and moth are described by black and pink lines, respectively. The bottom panels represent the time series data for 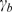 estimated by using the least-squares method. Dotted lines in the bottom panels represent 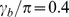 and 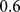. 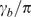 mainly exists within 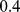 and 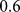.

**Figure 3:**
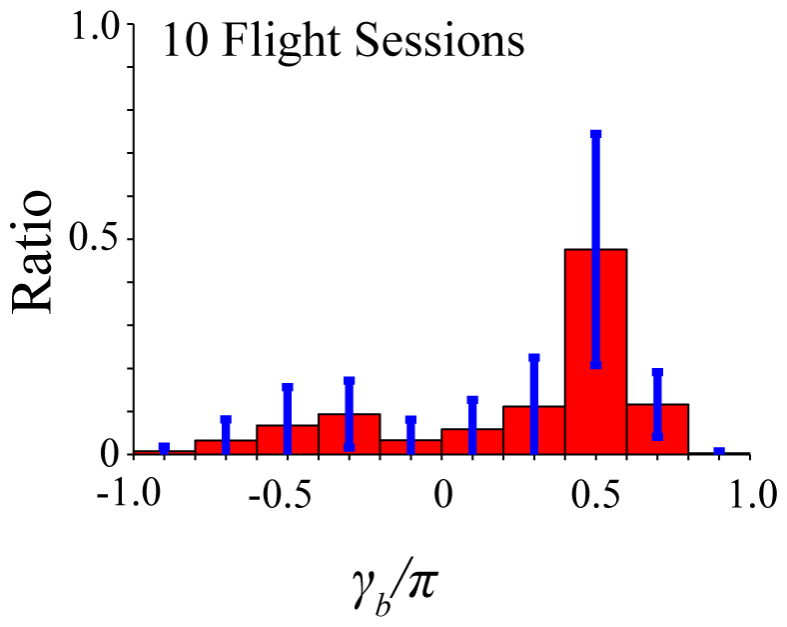
Normalized histogram of 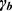 estimated from experimental data. This histogram was calculated with the bin size of 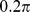, using the time series data for 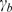 obtained from 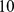 flight sessions of successful prey capture by 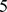 bats. Blue bars represent the standard deviations in each bin. The histogram of 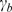 has an obvious peak in the bin of 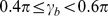.

Figure 2 represents the flight paths of a bat and a moth in the horizontal plane of the chamber, and the time series data for 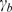 during 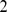 different flight sessions. In each session, different bat and moth individuals were used; 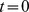 s corresponds to the time when the bat captured the moth. As shown in Figure 2, 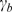 mainly exists between 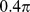 and 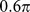. Figure 3 shows a normalized histogram of 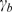 with the bin size of 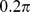 obtained from 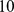 flight sessions of successful prey capture by 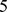 bats in a previous study [18], [21]. To empirically verify the result of the numerical simulation that 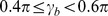 is a suitable range for prey capture, the 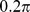 bins for 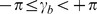 were used in this histogram. There is an obvious peak in the bin of 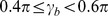.

Thus, the distribution of 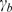 estimated from the experimental data is consistent with the optimal value derived by the numerical simulation shown in Figure 1C and D.

### Parameter Estimation: The Pursuit of Two Moths by a Bat

We examined the echolocation behavior of a bat towards 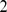 moths, based on the previous experimental data [24]. In the experiments, 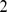 moths were simultaneously provided in the flight chamber, and a bat captured one of these moths. Video and sound recordings were made, following the same procedures described above for a single bat and a single moth. 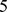 flight sessions with successful prey capture were obtained using 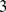 bats, and the flight paths of a bat and 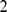 moths in the horizontal plane of the chamber were calculated as (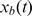, 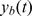), (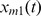, 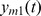), and (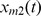, 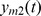) by analyzing the video recordings. Here, (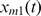, 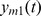) represents the flight path of the moth captured by a bat, and (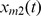, 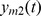) represents the flight path of the other moth.

The mathematical model for Equation 3 was extended to include pursuit behavior by a bat towards 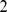 moths, as follows:
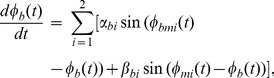
(11)where



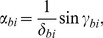
(12)

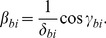
(13)



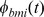
 (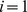, 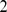) is the angle from the bat to the 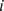
th moth, and 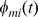 is the flight angle of the 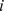
th moth. The parameters 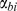 and 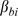 (or 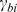 and 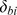) represent the way in which 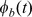 is affected by the flight path of the 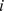
th moth.

Using the flight paths of each animal examined in the previous laboratory experiments [24], the parameters 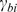 and 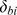 in Equations 12 and 13 were estimated. First, the values of 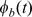, 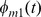, 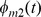, 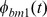, 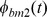, and 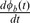 were calculated according to Equations 7–10. Second, Equation 11 was transformed to 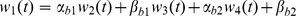 with 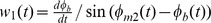, 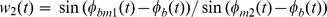, 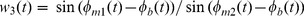, and 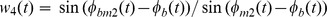. Third, the parameters 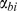 and 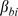 (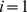, 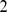) in Equation 11 were estimated at each time 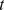, by applying the least-squares method to the neighboring 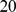 sets of 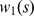, 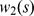, 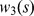, and 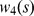 with 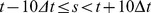. Finally, the parameters 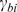 and 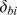 were then calculated using Equations 12 and 13.

**Figure 4:**
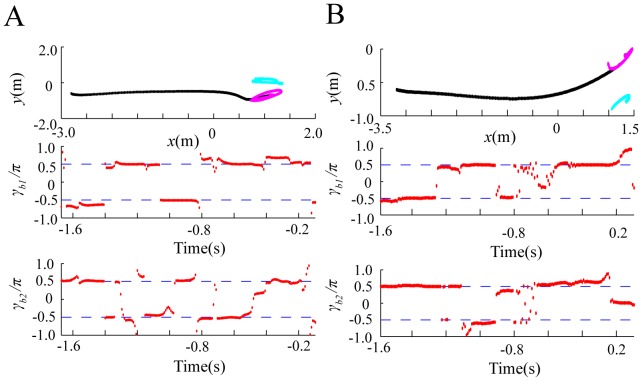
Parameter estimation of 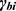 (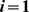, 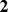) in Equations 12 and 13, from 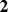 different flight paths of a bat and 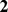 moths. These 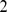 paths were experimentally obtained from video recordings in the flight chamber, using the same bat individual and different moth individuals. The top panels represent the flight paths of a bat and 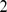 moths in the horizontal plane of the chamber, and the middle and bottom panels represent the time series data for 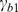 and 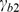. Note that the bat captured the first moth but not the second moth, during each session. In the top panels, the flight paths of the bat, the first moth, and the second moth are given by black, pink, and light blue lines, respectively. In the middle and bottom panels, dotted lines describe 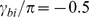 and 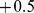 (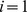, 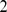). 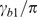 and 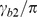 mainly exist around 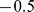 and 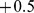.

Figure 4 shows the flight paths of each animal in the horizontal plane, and the time series data for 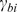 (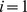, 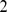). It can be seen that 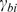 switches between 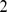 states of 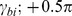 and 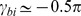 (the middle and bottom panels of Figure 4). In the 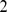 sessions shown in Figure 4, the same bat individual and different moth individuals were used.

**Figure 5:**
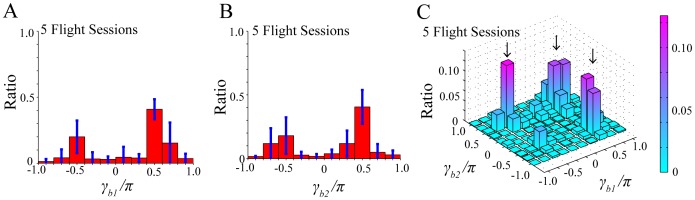
Normalized histograms of 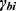 (*i* =  1, 2) obtained from experimental data, i.e., video recordings of 5 flight sessions of successful prey capture by 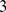 bats. (A) and (B) Normalized histograms of 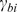 (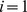, 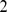) with standard deviations (blue bars) with the bin size of 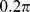. Each histogram has 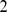 obvious peaks in the bins of 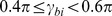 and 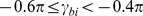, and the peak in 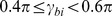 is higher than that in 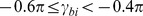. (C) Normalized histogram of 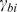 (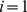, 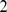) in the 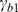-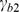 plane. There are 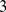 peaks around (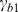, 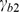) 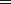 (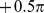, 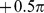), (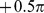, 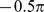), and (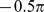, 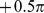) corresponding to different pursuit strategies of the bat towards 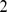 moths. The first peak represents simultaneous pursuit of both moths, the second peak represents selective pursuit of the first moth, and the third peak represents selective pursuit of the second moth.

The normalized histogram of 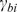 (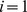, 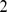) with the bin size of 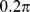 was taken from 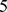 flight sessions of successful prey capture by 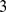 bats. As shown in Figure 5A and B, each histogram has 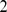 obvious peaks in the bins of 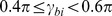 and 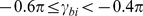; moreover, the peak in the bin of 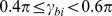 is higher than that in the bin of 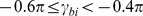. Here, 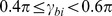 means that a bat approached the 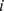
th moth, and 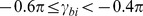 means that a bat flew away from the 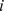
th moth: namely, whereas 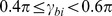 corresponds to a suitable value for a bat to capture a single prey in the numerical simulation, 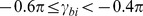 corresponds to the value for a bat not to capture the prey (Figure 1C and D). The origins of the 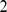 peaks are explained by the existence of 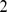 moths; if the 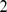 moths are positioned in different directions from the bat, the bat has to choose one of them and fly away from the other. Figure 5C shows the normalized histogram of 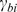 in the 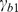-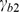 plane. There are 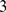 peaks around (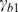, 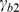) 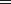 (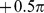, 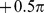), (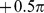, 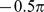), and (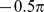, 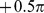). The peak around (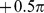, 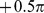) corresponds to simultaneous pursuit of both moths, the peak around (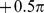, 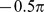) corresponds to selective pursuit of the first moth, and the peak around (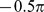, 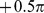) corresponds to selective pursuit of the second moth. Thus, the histogram of 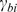 in the 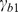-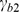 plane obtained from experimental data interprets the different types of spatial awareness shown by an echolocating bat towards 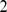 prey.

## Discussion

### Validity of the Present Mathematical Model

In the present mathematical model, Equation 3 describes the effects of 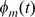 and 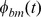 on 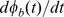. Numerical simulation using our model of Equations 3 and 4 has theoretically demonstrated that the effect of 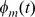 is also important for successful capture of a single prey by a bat, corresponding to relatively high 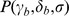 within 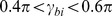, and much higher 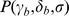 around 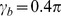 (Figure 1C and D). Note that, in the numerical simulation, the value of 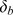 was restricted in 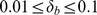, because the value of 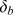 estimated from the experimental data was mainly distributed in that region. Moreover, the parameters 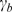 and 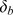 in Equations 5 and 6 were estimated on the basis of experimental data for the flight paths which were recorded by high-speed cameras in the flight chamber. The normalized histogram of 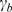 obtained from the experimental data had an obvious peak in the bin of 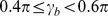 (Figure 3). This agreement between the numerical simulation and parameter estimation suggests that the bat chooses an effective flight path for successful prey capture by using 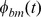 and 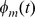, because Equation 6 with 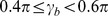 represents nonzero 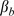, except for the case of 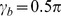. By contrast, 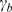 estimated from the experimental data sometimes takes a different value from 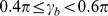 (the bottom panels of Figure 2). This is inconsistent with the results of our numerical simulation shown in Figure 1C and D. Further studies are required to investigate origin of this inconsistency, by considering other aspects of bat’s and prey’s behavior. For example, some species of insects detect the ultrasound emitted by bats, and thereby avoid being eaten [25], [26]. In such an interactive situation, the dynamics of prey’s escape behavior is important in determining how the bat precisely pursues the prey.

For a bat and 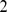 moths, the parameters 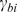 and 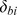 (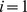, 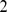) in Equations 12 and 13 were also estimated by using the video recordings obtained in the flight chamber. As shown in Figure 5C, the histogram of 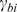 (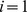, 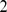) in the 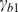-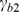 plane had 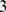 obvious peaks around (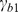, 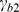) 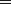 (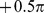, 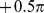), (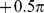, 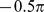) and (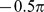, 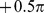). This result suggests that the bat’s strategy of pursuit towards 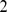 prey can be understood on the basis of (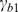, 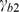), because the 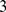 peaks indicate the different types of spatial awareness shown by an echolocating bat; (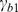, 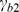) 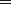 (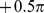, 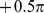) corresponds to simultaneous pursuit of both moths, (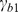, 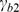) 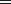 (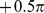, 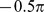) corresponds to selective pursuit of the first moth, and (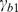, 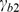) 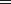 (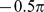, 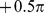) corresponds to selective pursuit of the second moth. From theoretical point of view, important future works include numerical simulation of the success rate of prey capture for the case of one bat and two prey by changing the parameter values of 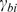 and 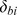 (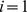, 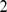) in Equations 12 and 13, which can be compared with the results of the parameter estimation shown in Figure 5.

In summary, the present mathematical model quantitatively describes a bat’s echolocation strategy, as well as qualitatively elucidates the dynamics of bat’s attention to multiple prey. Our study is the first to evaluate a bat’s flight path during multiple prey pursuit, by using a mathematical model.

### Behavioral Meanings of 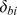 Estimated from Experimental Data

To examine how the parameters 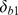 and 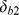 in the mathematical model explain the behavioral aspects of bat’s pursuit towards 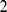 prey, the estimated values of 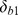 and 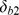 were compared with experimental data for the emission angles of ultrasound pulses.

First, the sound pressure levels at various angles from the bat were estimated by using the sound recordings obtained from the microphone array system [18], and the angle of the maximum sound pressure level was defined as the emission angle of the ultrasound pulses, 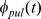. Second, 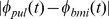 (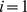, 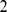) was calculated, by using 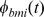 estimated from the video recordings. Here, 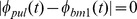 means that the bat emitted pulses towards the first moth (i.e., the moth captured by the bat), and 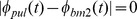 means that the bat emitted pulses towards the second moth (i.e., the moth not captured by the bat). In other words, we can estimate towards which moth the bat emitted ultrasound pulses, on the basis of the value of 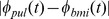 obtained from the sound and video recordings. In addition, 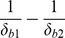 was calculated from 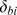 (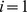, 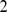). As shown in Equations 11, 12 and 13, 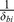 (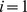, 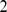) represents the magnitude of the effect from the 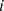
th moth on 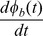. Therefore, it is expected that, the sign of 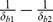 is positive (or negative), when the effect of the first moth (or the second moth) on 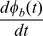 is dominant.

**Figure 6:**
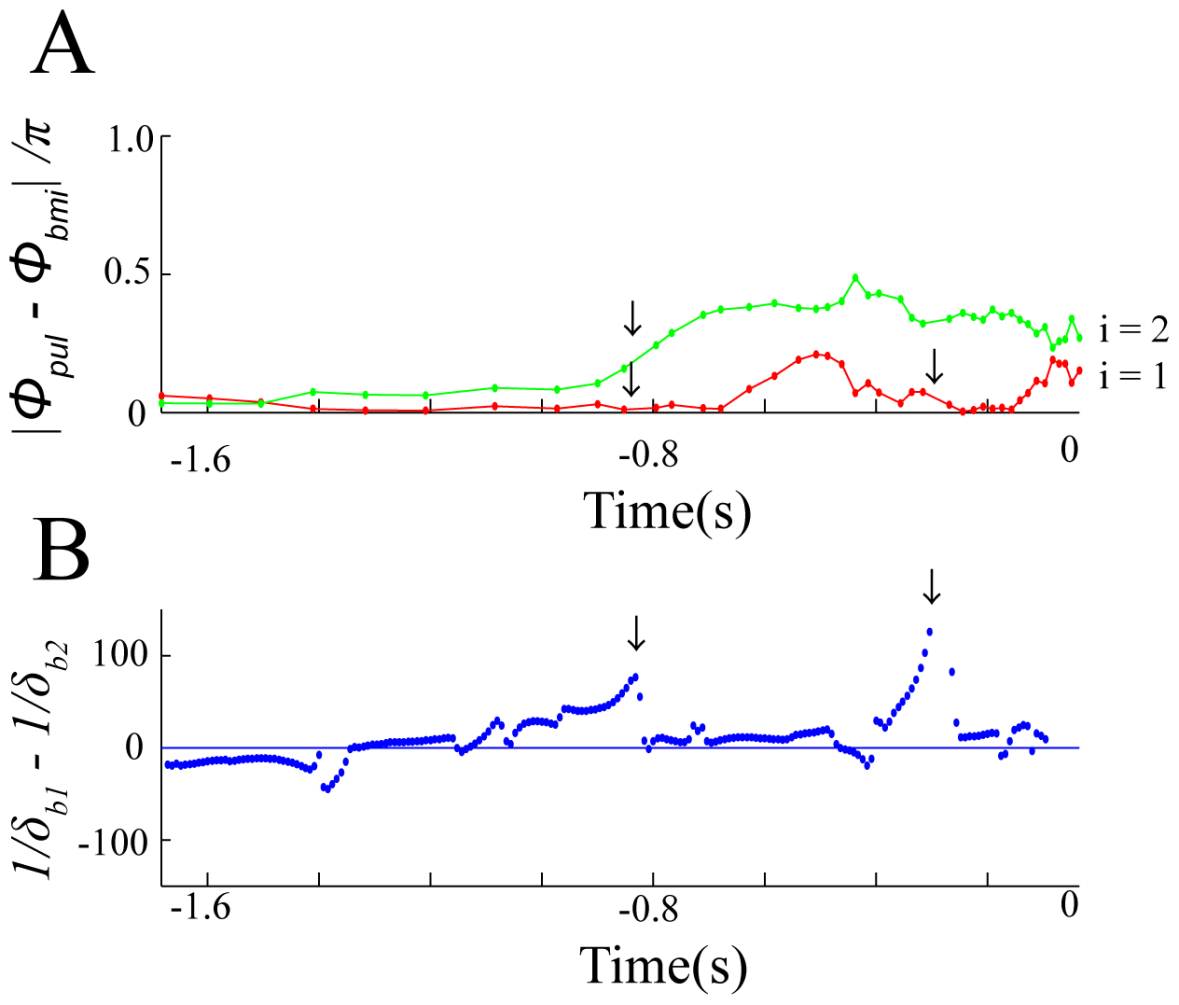
Comparison of 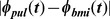 (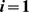, 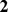) with 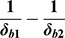. Time series data for 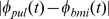 (A) and for 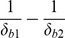 (B) were obtained from the flight session shown in Figure 4A. In Figure 6A, 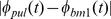 and 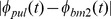 are described by red and green lines, respectively. The dynamics of 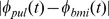 (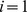, 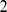) is qualitatively explained by the dynamics of 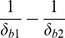, as emphasized by arrows: when 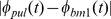 was closer to zero, 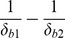 had a large positive value, corresponding to an increase in the bat’s attention towards the first moth. For instance, when 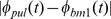 was close to zero and 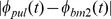 became larger, 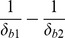 had a large positive value (see the arrows around 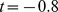 s in Figure 6A and B); when 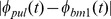 became closer to zero, 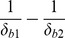 had a large positive value (see the arrows around 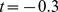 s in Figure 6A and B). Note that 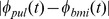 was calculated by using experimental data obtained from sound and video recordings, while 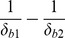 was estimated by fitting the mathematical model of Equation 11 to experimental data obtained from video recordings.

Figure 6 represents the time series data for 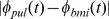 (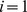, 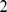) and for 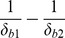, which were obtained from the flight session shown in Figure 4A. The dynamics of 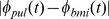 (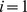, 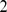) was qualitatively explained by the dynamics of 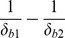: namely, when 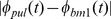 was closer to zero, 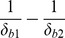 had a large positive value, corresponding to an increase in the bat’s attention towards the first moth. For instance, when 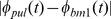 was close to zero and 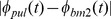 became larger, 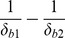 had a large positive value (see the arrows around 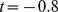 s in Figure 6A and B). This suggests that, if the bat was targeting its pulses towards the first moth rather than the second moth, its attention towards the first moth was increasing. Moreover, when 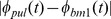 became closer to zero, 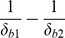 had a large positive value (see the arrows around 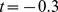 s in Figure 6A and B). This suggests that, if the bat was more precisely targeting its pulses towards the first moth, its attention towards the first moth was increasing. These agreements between the dynamics of 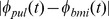 and 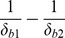 indicate that the dynamics of the emission angle of the ultrasound pulses can be understood by using 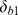 and 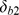 in our present mathematical model.

Thus, the present mathematical model contributes to qualitative evaluation of interaction between a bat’s dynamical flight and echolocation strategies.

### Possible Applications of the Mathematical Model

The present mathematical model may be extended to evaluate the natural foraging behavior of bats. To achieve effective pursuit in natural habitats, bats must sense the echoes reflected from multiple prey, and choose a suitable flight path to capture the most prey in the least amount of time. Moreover, several bat individuals simultaneously gather for foraging, and therefore the interactions between bats are important. Future works as an extension of the present model include experimental and theoretical evaluation of the foraging behavior of bats in the field, as well as modelling acoustic interactions between bats. Such studies will facilitate an understanding of the bat strategy for choosing a suitable flight path in a more complex environment, consisting of many bat and prey individuals.

Bats actively use auditory information to pursue prey, and can interact each other via ultrasound. Regarding control and robotics, the present model of echolocating bats will be applicable to autonomous distributed control of multiple agents. In particular, cooperative control of echolocating agents will be useful for achieving human tasks that are difficult to perform in the dark. The mathematical model of echolocating bats can be extended to such a control method for artificial agents in the engineering field, by mimicking the unique sensory systems of bats.
